# The Green Leaf Volatile (Z)-3-Hexenyl Acetate Is Differently Emitted by Two Varieties of *Tulbaghia violacea* Plants Routinely and after Wounding

**DOI:** 10.3390/plants11233305

**Published:** 2022-11-29

**Authors:** Alessandro Frontini, Luigi De Bellis, Andrea Luvisi, Federica Blando, Samar Min Allah, Rosanna Dimita, Carlo Mininni, Rita Accogli, Carmine Negro

**Affiliations:** 1 Department of Biological and Environmental Sciences and Technologies (DiSTeBA), Salento University, Via Prov.le Lecce-Monteroni, 73100 Lecce, Italy; 2 Institute of Sciences of Food Production (ISPA), National Research Council (CNR), Research Unit of Lecce, Via Prov.le Lecce-Monteroni, 73100 Lecce, Italy; 3 Ortogourmet Società Agricola S.r.l., S.C. 14 Madonna delle Grazie, 74014 Laterza, Italy

**Keywords:** acetylated C_6_ aldehyde, wounding, plant-defense response, induced plant volatiles

## Abstract

While studying aromas produced by the edible flowers of *Tulbaghia violacea*, we noticed a different production of (Z)-3-Hexenyl acetate (a green-leaf volatile, GLV) by purple (var. ‘Violacea’) and white (var. ‘Alba’) flowers. The white *Tulbaghia* flowers constantly emits (Z)-3-Hexenyl acetate, which is instead produced in a lower amount by the purple-flowered variety. Thus, we moved to analyze the production of (Z)-3-Hexenyl acetate by whole plants of the two varieties by keeping them confined under a glass bell for 5 h together with a SPME (Solid Phase Micro Extraction) fiber. Results show that six main volatile compounds are emitted by *T. violacea* plants: (Z)-3-Hexenyl acetate, benzyl alcohol, nonanal, decanal, (Z)-3-Hexenyl-α-methylbutyrate, and one unknown compound. By cutting at half-height of the leaves, the (Z)-3-Hexenyl acetate is emitted in high quantities from both varieties, while the production of (Z)-3-Hexenyl-α-methylbutyrate increases. (Z)-3-Hexenyl acetate is a GLV capable of stimulating plant defenses, attracting herbivores and their natural enemies, and it is also involved in plant-to-plant communication and defense priming. Thus, *T. violacea* could represent a useful model for the study of GLVs production and a ‘signal’ plant capable of stimulating natural defenses in the neighboring plants.

## 1. Introduction

*Tulbaghia violacea* Harv. (1837) is a perennial-rhizomatous plant native to South Africa, now used as an ornamental plant and variously distributed throughout the world. Linnaeus coined the genus in honor of governor the Dutch Cape Colony, Ryjk Tulbagh (1699–1771), who sent him numerous specimens of the local flora [1]. The species name naturally refers to the purplish color of the flowers. Both the leaves and the flowers have a garlic-like flavor and are used for flavoring salads and other dishes; in fact, common *T. violacea* names recall garlic: society garlic, sweet garlic, and wild garlic. The popularity of the species is linked to the mistaken belief that its consumption does not leave an unpleasant breath as happens after garlic consumption [2]. A common characteristic between plants of the *Tulbaghia* and *Allium* genera is a ‘garlic’ smell that is produced when leaves or rhizomes are damaged; the precursors of the typical odor are cysteine-derived amino acids present in the cytoplasm, and S-(methylthiomethyl)-cysteine-4-oxide (marasmin) in the case of *Tulbaghia* and S-allyl-cysteine sulfoxide (alliin) in the case of *Allium*. When the tissues are damaged, a C-S lyase, present in the vacuoles of the cells, comes into contact with marasmin (or alliin), cleaving the compound into thiosulphinate marasmicin (or allicin) [3]. Marasmicin is not stable and decomposes giving origin of other volatile sulfur compounds, especially if *Tulbalghia* organs or tissues are subjected to heat treatment [4,5].

We became interested in *T. violacea* in the course of a study focusing on volatile compounds emitted by edible flowers and coincidentally noticed differences in the emission of volatile compounds between white and purple flowers of different varieties of *T. violacea* (var ‘Violacea’ and ‘Alba’); our attention was drawn to the emission of (Z)-3-Hexenyl acetate (Z3HAC), a compound part of the green leaf volatiles (GLVs), emitted by plants both constitutively and in response to wounding and attacks by herbivores [6], which are also involved in triggering plant-defense responses via jasmonic acid [7,8].

GLVs, with their typical smell of freshly cut grass, are produced by almost all plants and constitute an important group of volatile organic compounds emitted by plants. They are C6 aldehydes and acetylated C_6_ aldehydes deriving from the lipid oxidation of fatty acids in the oxylipin pathway (common to jasmonic acid) and are rapidly emitted as a result of tissue damage due to biotic or abiotic stress [6]. GLVs as Z-3-Hexenal, (E)-2-Hexenal, Z-3-Hexenol, and Z3HAC represent communication and defense signals that can activate defensive reactions in the same plant and neighboring plants against herbivores and pathogens, and repel or attract herbivores and their natural enemies, developing in addition some toxicity towards bacteria and fungi [6,9]. In addition, GLVs represent a pleasant powerful green-note/fruity-aroma component of apples and tropical fruits [10,11] so as to be efficiently employed e.g., in a recipe for the artificial imitation of mango flavor [12].

The aim of this study was to find differences of GLVs, if any, between white and purple flowers of *T. violacea*, both in normal conditions and in response to wounding.

## 2. Results

### 2.1. Main Volatile Compounds Emitted by Tulbaghia Flowers

**Figure 1:**
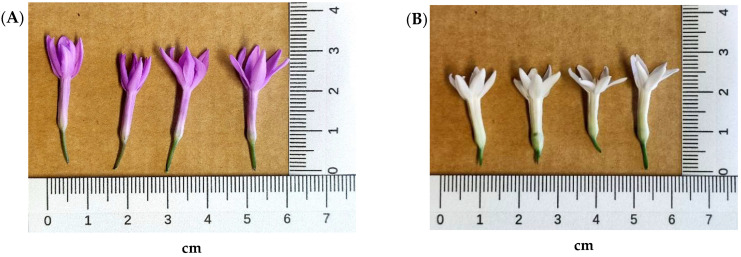
Purple and white flowers of *T. violacea* from the two varieties. (**A**): var. ‘Violacea’: (**B**): var. ‘Alba’.

**Figure 2:**
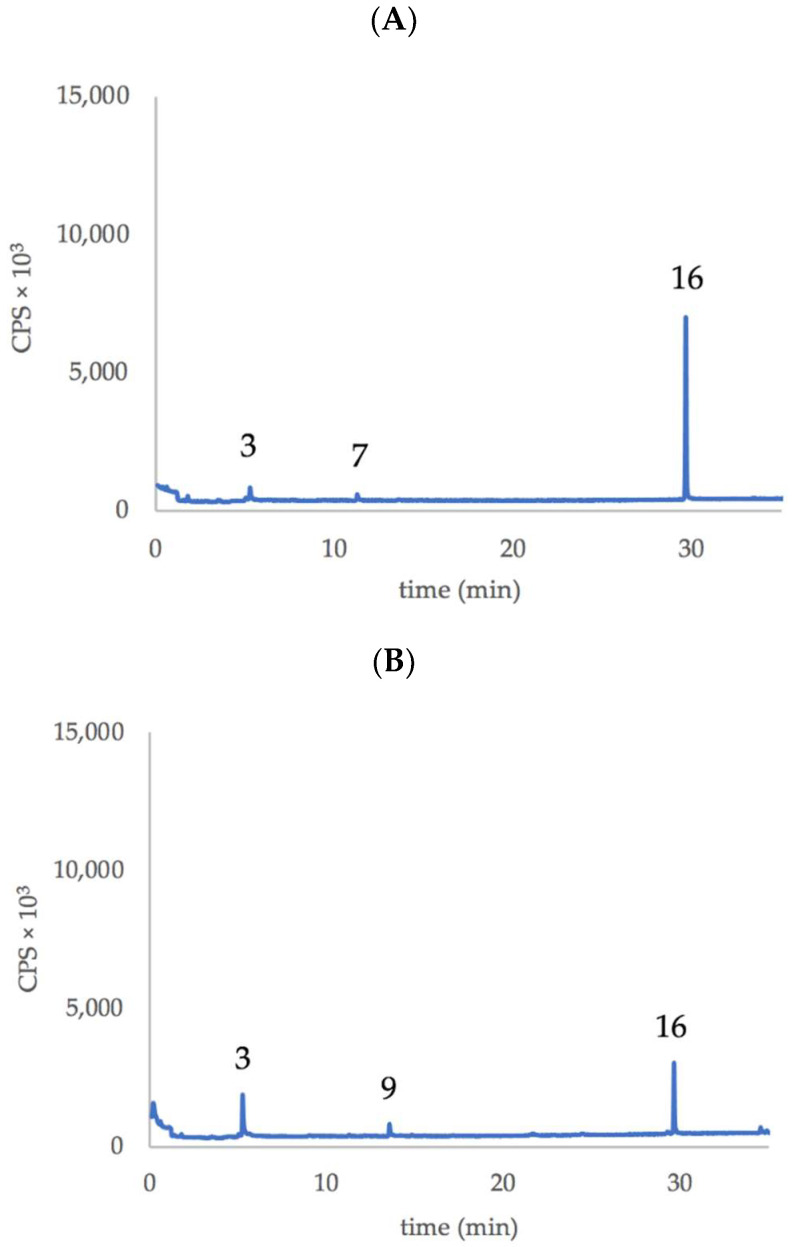
Representative chromatograms showing the main VOCs emitted by the flowers *T. violacea*. (**A**) purple flowers of var. ‘Violacea’, (**B**) white flowers of var. ‘Alba’. Numbers indicate the compounds listed in Table 1.

Edible *T. violacea* flowers (Figure 1) show an emission of similar volatile compounds but in different ratio depending on the variety/flower color. In fact, in both flower types, we detected mainly the emission of 2,4,5,7-Tetrathiaoctane and Z3HAC in a ratio, calculated as a peak area, of approximately 4:1 for the purple flowers and of 2:1 for white flowers. Table 1 and Figure 2 summarize more clearly the identified volatile organic compounds (VOCs) emitted by flowers of the two varieties of *Tulbaghia*.

**Table 1:** Main VOCs emitted by *T. violacea* flowers var. ‘Violacea’ and ‘Alba’, purple and white flowers, respectively. Numbering of volatile compounds respects the order of elution considering the set of compounds identified after the analysis of flowers and of the whole plants (see Table 2) so as to keep the numbering univocal. The values given are the averages of three repetitions; standard-error values are not reported but were all values within 9%.

No.	RI	Compound Name	Peak Area %	
			Purple	White
**1**	889	2,4-Dithiapentane	0.51	**-**
**3**	991	(Z)-3-Hexenyl acetate	9.85	22.15
**7**	1134	2,3,5-Trithiahexane	3.91	1.68
**9**	1175	Benzyl 3-hydroxypropanoate	1.32	5.54
**10**	1185	(Z)-Butanoic acid, 3-hexenyl ester	0.50	0.93
**15**	1501	4-Formyl-3,5-dimethyl-1H-pyrrole-2-carbonitrile	**-**	0.56
**16**	1528	2,4,5,7-Tetrathiaoctane	44.5	46.31

Retention index (RI) was obtained as a function of the retention times of a series of C_8_–C_20_ alkanes (for more details see Section 4).

**Table 2:** VOCs emitted by whole plants of *T. violacea* and captured by SPME fiber in 5 h wounding indicates that 50% of the leaves were cut at half of their height (**+**: presence; **-**: absence/trace amount). Numbering of volatile compounds respects the order of elution considering the set of compounds identified after the analysis of flowers and of the whole plants (see Table 1) so as to keep the numbering univocal.

No.	RI	Compound Name	Whole Plant			
			Var. ‘Violacea’		Var. ‘Alba’	
			Control	Wounding	Control	Wounding
**2**	934	Benzaldehyde	**+**	**+**	**+**	**+**
**3**	991	(Z)-3-Hexenyl acetate	**+**	**+**	**+**	**+**
**4**	1038	Benzyl alcohol	**+**	**+**	**+**	**+**
**5**	1098	(Z)-3-Hexen-1-ol,propanoate	**-**	**+**	**-**	**+**
**6**	1110	Nonanal	**+**	**+**	**+**	**+**
**8**	1170	(Z)-1,7-Octadiene-3,6-diol,2,6-dimethyl	**-**	**+**	**-**	**+**
**10**	1185	(Z)-Butanoic acid, 3-hexenyl ester	**-**	**+**	**-**	**+**
**11**	1206	Decanal	**+**	**+**	**+**	**+**
**12**	1215	(Z)-1,6-Octadien-3-ol,3,7-dimethyl-formate	**-**	**+**	**-**	**+**
**13**	1226	(Z)-3-Hexenyl-α-methylbutyrate	**+**	**+**	**+**	**+**
**14**	1275	(Z)-Hex-3-enyl(E)-2-methylbut-2-enoate	**-**	**+**	**-**	**+**
**16**	1528	2,4,5,7-Tetrathiaoctane	**+**	**+**	**-**	**+**

Retention index (RI) was obtained as a function of the retention times of a series of C_8_–C_20_ alkanes (for more details see Section 4).

The relevant finding is the diverse emission ratio between Z3HAC and 2,4,5,7-Tetrathiaoctane by the two different flowers (Figure 2), suggesting a different pattern between the two varieties of *Tulbaghia* regarding the emission of GLVs and the production of metabolites derived from marasmicin. In fact, 2,4,5,7-Tetrathiaoctane and 2,3,5-Trithiahexane are the result of flower-peduncle cutting (about 5–6 flowers make 1 g), manipulation and subsequent heating of the sample and fiber during the analytical procedure. The damaged (or homogenized) *Tulbaghia* tissues allow the contact between marasmin and a C-S lyase that cleaves the compound, yielding (methylthio)methane-sulfenic acid, a short-lived compound that condenses into marasmicin (2,4,5,7-tetrathiaoctane 4-oxide), which in turn is transformed to 2,4,5,7-Tetrathiaoctane and 2,3,5-Trithiahexane, particularly upon boiling of *T. violacea* rhizomes [4,5].

In addition to the previous results, a semiquantitative determination of the Z3HAC emitted by *Tulbaghia* flowers confirms that ‘Alba’ flowers emit approximately 2.5 times more than those of the var. ‘Violacea’ per g fresh weight (Table 3).

### 2.2. Volatile Compounds Emitted by Whole Tulbaghia Plants

**Figure 3:**
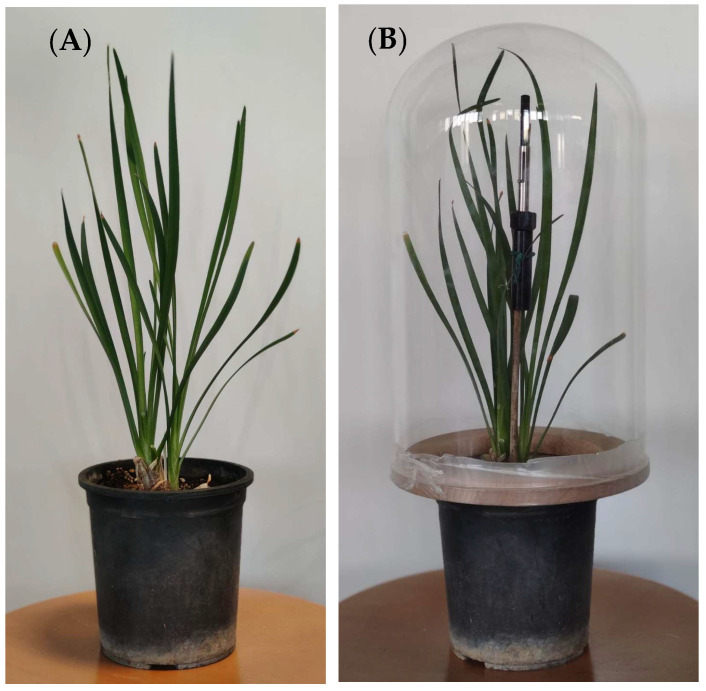
*T. violacea* plant in a pot (**A**) and under a glass bell in the presence of SPME fiber (**B**).

Thus, the next step was to check whether the emission of Z3HAC by whole *Tulbaghia* plants varied according to variety and/or wounding treatment; for this purpose, we proceeded to collect, via SPME fibers, the volatile compounds emitted by plants kept for several hours inside a glass bell (Figure 3).

**Figure 4:**
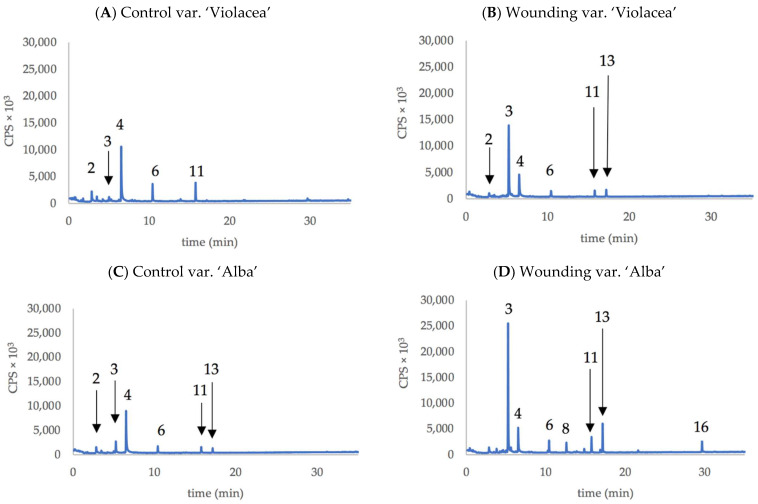
Chromatograms of main VOCs produced by the whole plant of purple-flowered *T. violacea* ((**A**): Control; (**B**): after wounding) and white-flowered *T. violacea* ((**C**): Control; (**D**): after wounding). Numbers indicate the compounds listed in Table 2.

Table 2 presents the compounds released by whole plants of the two varieties of *Tulbalghia* before and after wounding (50% of the leaves cut at half of their height). None of the 16 compounds detected appeared to be variety-specific, but five of them appeared to be produced specifically as a result of wounding, although in a very low amount: (Z)-3-Hexen-1-ol,propanoate, (Z)-1,7-Octadiene-3,6-diol,2,6-dimethyl, (Z)-Butanoic acid,3-hexenyl ester, (Z)-1,6-Octadien-3-ol, 3,7-dimethyl-formate, and (Z)-Hex-3-enyl(E)-2-methylbut-2-enoate. In fact, of these, only (Z)-1,7-Octadiene-3,6-diol,2,6-dimethyl (compound no. 8) appeared evident in representative chromatograms of volatile compounds emitted by plants of the two varieties, both control plants and after wounding (Figure 4B,D).

In both *Tulbaghia* varieties, there was an increase in the production of Z3HAC and of some related compounds, such as (Z)-3-Hexenyl-α-methylbutyrate (compound 13 in Figure 4).

The main volatile compounds, such as benzyl alcohol, nonanal, and decanal, also persisted, while most of the sulfur compounds deriving from marasmin, which are emitted by flowers, were not detected in this analysis, except in trace amounts. This is because, in this case, the ratio of damaged tissues/whole biomass was very small.

Regarding the production of Z3HAC, the wounding treatment resulted in a significant increase in emission in both *Tulbaghia* varieties, over 4000% for the ‘Violacea’ and about a quarter for the ‘Alba’, with the latter, however, emitting about twice as much following wounding treatment (Table 4).

## 3. Discussion

The Z3HAC, which are derived from C_6_-aldehydes as (Z)-3-Hexenal and *n*-Hexenal, are metabolites produced from oxylipins (oxygenated compounds derived from fatty acids) along one of the two main oxylipin-pathway branches regulated by hydroperoxide lyase [13,14,15]. The other oxylipin-pathway branch, key-enzyme, allene-oxide-synthase, gives origin to jasmonates which are involved in a huge amount of defense responses [16,17,18].

We focused our work by finding that *T. violacea* plants emits significant amounts of Z3HAC; such compound was easily detected employing a solid-phase, micro-extraction methodology, both from the flowers and from the whole plant. The emission was lower in the purple flowers than in white flowers, whereas, by cutting at about half-height of the plant leaves, the response of the two *Tulbaghia* varieties was an emission of Z3HAC of approximately 10 to 50 times (up to 260 and 440 ng plant^−1^ h^−1^, respectively), compared with the non-wounding plants (Table 4). In addition, the production of the related compound cis-3-Hexenyl-α-methylbutyrate increased slightly (Figure 4).

More than fifteen years ago, Z3HAC and (Z)-3-Hexenol were identified as volatile compounds present in very low amounts (0.1–0.6%) in oil obtained from aerial parts of *T. violacea* by Pino et al. [19], but thereafter, such presence was not associated with GLVs. The only somehow related reference is the use of oil extract of *T. violacea* to strongly repel aphids [20].

An increase in the emission of Z3HAC as a result of wounding or mechanical damage was also detected in other plants. For instance, in corn plants, Z3HAC was released in greater quantities by plants damaged by insects (*Elasmopalpus lignosellus* larvae) than by plants not damaged or damaged manually (using a needle); notably, the volatile compound was released in large quantities after 24 and 96 h after treatment [21]. Instead, an increase of 7 or 18 times in the amount of Z3HAC emitted was observed using a SPME fiber for soybean leaves or pods mechanically damaged (with an entomological needle), in comparison to tissues damaged by insects (*Rhyssomatus nigerrimus*, Mexican weevil of soy) or healthy plants [22].

In addition to being involved in the internal communication of the plant and with neighboring plants as a trigger for immune-defense responses [6], Z3HAC appeared to be an attractive compound for both beneficial and harmful insects.

Concerning the attraction of beneficial insects, Arimura et al. [23] and Ozawa et al. [24] have observed that *Lotus japonica* plants infested by the spider mites *Tetranychus urticae* start to emit several volatiles, including a significant amount of Z3HAC and methyl salicylate, attracting *Phytoseiuus persimilis* (a predatory mites). Chehab et al. [13] indicated that Z3HAC is the predominant compound in the volatile blend emitted by Arabidopsis after aphid attack that mediates the attraction of aphid natural enemies (parasitoid wasp *Aphidius colemani*); Xavier et al. [21] revealed that the parasitoid, *Trichogramma pretiosum*, is attracted by VOCs released (mainly Z3HAC) by corn plants damaged by the larva of the insect, *Elasmopalpus lignosellus*, while Hegde et al. [25] reported that cotton plants, emitting a volatile blend including Z3HAC, repels the cotton aphid (*Aphis gossypii*). Also, it was described that Z3HAC is one of the major volatile components emitted by wheat seedlings, particularly when infested with aphids, and that spraying it on wheat field determines an attraction of ladybugs, reducing the abundance of aphids [26]. Interestingly, it is also demonstrated by electroantennograms and Y-tube olfactometer experiments that Z3HAC alone or cis-jasmone alone are attractive to *Campoletis chlorideae*, the parasitoid of the cotton bollworm, *Helicoverpa armigera*, but not the mixture of the two compounds [27].

Unfortunately, Z3HAC attracts harmful insects, such as the cutworm *P. saucia* [28] and the *Holotrichia oblita* beetle [29], but this phenomenon turns to be a vantage of pheromone traps. One case is the attraction of *Plutella xylostella* (cruciferous moth) which, in field experiments, were caught in greater numbers with synthetic-sex-pheromone traps containing Z3HAC alone or a mixture of Z3HAC, (Z)-3-Hexen-1-ol, and (E)-2-Hexenal, compared to sex pheromone alone [30]; other cases are the efficacy of sex-pheromone traps in a pear orchard where more males of *Grapholita molesta* (oriental fruit moth) are captured with traps containing pheromone plus Z3HAC then with the addition of 1-undecanol [31], and the increased catches of *Agriotes brevis* (click beetles, harmful to maize) when a mix of Z3HAC, methyl benzoate, (Z)-3-Hexenol, and methyl salicylate is added to the synthetic pheromone [32]. In field tests, Z3HAC is also a specific attractant of female *Holotrichia parallela* (dark black chafers, an important pest of several crops), enabling the capture of six times more insects than the pheromone-L-leucine-methyl ester; on the contrary, for the capture of *H. parallela* males, pheromone is found to be about three times more effective [33].

On the other hand, it has been observed that plants with higher emission levels of Z3HAC can attract female insects for oviposition: in Xin et al. [34], the insect, *Empoasca vitis*, chooses tea-plants varieties with higher Z3HAC emission levels; in another study [35], it is observed that *Cnaphalocrocis medinalis* has a negligible impact on corn whether there are rice plants in the vicinity, speculating that this could be due to different emission levels of VOCs, including Z3HAC. This feature could be exploited by the employment of ‘bait’ plants, removing them from the field after the oviposition stage, or using them for monitoring and detecting the abundance of phytophagous.

Finally, a study by Najdabbasi et al. [36] addresses the potential role of Z3HAC in decreasing the severity of late blight, the most important fungal disease of potato plants; Z3HAC provides a protection to pre-exposed plants against five genotypes of *P. infestans*, reducing late-blight severity by around 70% in a very susceptible potato cultivar. Similarly, Ameye et al. [37] demonstrated that exposure of *Triticum aestivum* plants to Z3HAC (priming) before artificial infection with *Fusarium graminearum* reduced the incidence of the relative pathology of *Fusarium* head blight. They observe a significant reduction of both fungal biomass and spikelets showing necrotic lesions in preexposed plants and a different expression of jasmonic acid- and salicylic acid-responsive genes. This suggests new opportunities for sustainable control of fungal diseases through activation of plant immunity.

There are slight increases of (Z)-3-Hexenyl-α-methylbutyrate after wounding (Figure 4); a similar increase for this Z3HAC derivate (and for the simpler butyrate form) was disclosed by McCall et al. [38] for heavily damaged cotton seedlings (by five *Helicoverpa zea* caterpillars/plant).

Thus, the two varieties of *T. violacea*, particularly the ‘Alba’ variety, represent a tool for studying the bio-synthetic pathway leading to Z3HAC production as well as ‘signal’ organisms capable of stimulating natural defenses in neighboring plants. Additionally, the two varieties of *Tulbaghia* could be employed for screening insects attracted to or repelled by Z3HAC with the aim of developing effective, low-cost, biological-control protocols.

## 4. Materials and Methods

### 4.1. Plant Culture Conditions

Plants of *T. violacea* var. ‘Violacea’ and var. ‘Alba’ were obtained from the Ortogourmet company (Laterza, Taranto, Italy) in pots of 18 cm in diameter and then transferred and grown in pots of 10 cm in diameter in an unheated greenhouse from October to April (and for the rest of the year in the open air) in the Botanical Garden of the University of Salento, Lecce, Italy.

### 4.2. Analysis of Volatile Organic Compounds

The analyses were carried out by solid-phase micro extraction (SPME) methodology, as described previously by Negro et al. [39] and Dimita et al. [40]. *Tulbaghia* flowers were collected at full bloom and 5–6 of them (approximately 1g FW) were immediately sealed into 20 mL SPME vials (Agilent Technologies, Palo Alto, CA, USA) by metal screw-caps with pre-notched, Teflon-silicone septa. The vials were then placed at 40 °C for 10 min in a thermostatically controlled bath to allow the evaporation of the compounds; hereafter, a SPME syringe was inserted, and the fiber (50/30 µm Divinylbenzene/Carboxen/Polydimethylsiloxane, Supelco/Merck KGaA, Darmstadt, Germany), which was previously conditioned for 5 min at 235 °C in the gas-chromatograph injector, was exposed for 10 min to absorb the volatile compounds.

In a wounding experiment, whole plants with approximately 18–22 leaves and no flowers (both in the case of control plant and after wounding with 50% of the leaves cut at half their height) were confined under a glass bell, resting on a wooden base with a hole in the middle, so that the epigeal part of the plant could be isolated from the pot (Figure 4). Additionally, in order to avoid contaminating the SPME fiber with soil emissions, an aluminum foil was placed between the wooden base and the soil, surrounding the root collar. The SPME syringe (previously conditioned for 5 min at 235 °C in the gas-chromatograph injector) was tied in the middle of the bell to a stick and exposed for 5 h at room temperature (Figure 3).

Subsequently, the fiber was inserted into the injector port of a gas chromatography with a mass-spectrometry detector (Agilent 7890B coupled with MS single quadrupole Agilent 5977A), and the desorption of the volatile compounds performed at 235 °C for 4 min. At this point, the chromatographic run was started with an Agilent HP-5 ms column (30 m × 0.25 mm, 0.25 µm) (where temperature was raised from 60 °C to 230 °C with a constant increase of 3 °C/minute), with a helium (purity >99.999%) constant flow of 1.0 mL/min. Compounds were identified by library search and analytical standard if available. The mass spectrum of an unknown compound was searched in a data-processing system [41]. Substances with a score above 800, both for identity and purity, were putatively identified after comparing the detected compound with the one in the NIST Computational Chemistry Comparison and Benchmark database [41]. Retention index (RI) was obtained, as reported by Zhao et al. [42], being employed as a reference of the retention times of a series of C_8_–C_20_ alkanes separated under the GC-MS conditions mentioned above; the following formula was applied:

$$\mathrm{RI}=100\times n+\frac{100\;\left(t_{a}-t_{n}\right)}{t_{n+1}-t_{n}}$$

where, *t_a_* is the retention time of the unknown peak a; *t_n_* the retention time of *n*-alkane *Cn*; and *t_n_*_+1_ the retention time of *n*-alkane *C_n_*_+1_; *n* = carbon number of the alkane which elutes before the unknown peak a.

The semi-quantitative analysis of volatile compounds was carried out in the same experimental conditions, using known quantities of (Z)-3-Hexenyl acetate.

### 4.3. Statistics

All data were reported as the mean ± standard deviation (SD), with at least three replications for each sample. Statistical evaluation was conducted by Duncan’s test to discriminate among the mean values. All statistical analyses were performed using the software, Statistica (StatSoft, Tulsa, OK, USA).

## Figures and Tables

**Table 3:** Semi-quantitative determination of (Z)-3-Hexenyl acetate in white and purple flowers of T. violacea (ng/g fresh weight).

Flower	(Z)-3-Hexenyl Acetate (ng/g FW)
Purple	124.18 ± 6.28
White	314.56 ± 15.19

**Table 4:** Semi-quantitative determination of (Z)-3-Hexenyl acetate emitted after wounding by whole plants of white-flowered and purple flowered *T. violacea* (ng/plant in 5 h).

*Tulbaghia* Variety	(Z)-3-Hexenyl Acetate (ng/plant)		Increase (%)
	Control	Wounding	
‘Violacea’	28 ± 1	1300 ± 62	4463
‘Alba’	216 ± 10	2212 ± 109	926

## Data Availability

Not applicable. All results are included in the article.
